# Author Correction: Potent effects of dioscin against obesity in mice

**DOI:** 10.1038/s41598-020-74985-y

**Published:** 2020-10-22

**Authors:** Min Liu, Lina Xu, Lianhong Yin, Yan Qi, Youwei Xu, Xu Han, Yanyan Zhao, Huijun Sun, Jihong Yao, Yuan Lin, Kexin Liu, Jinyong Peng

**Affiliations:** 1 College of Pharmacy, Dalian Medical University, Western 9 Lvshunnan Road, Dalian, 116044 China; 2 Research Institute of Integrated Traditional and Western Medicine of Dalian Medical University, Dalian, 116044 China

Correction to: *Scientific Reports* 10.1038/srep07973, published online 22 January 2015

This Article contains an error.

In Figure 1D, the image is incorrect for the Oil Red HFD+Dio 20 panel. The correct Figure appears below as Figure 1.

**Figure 1:**
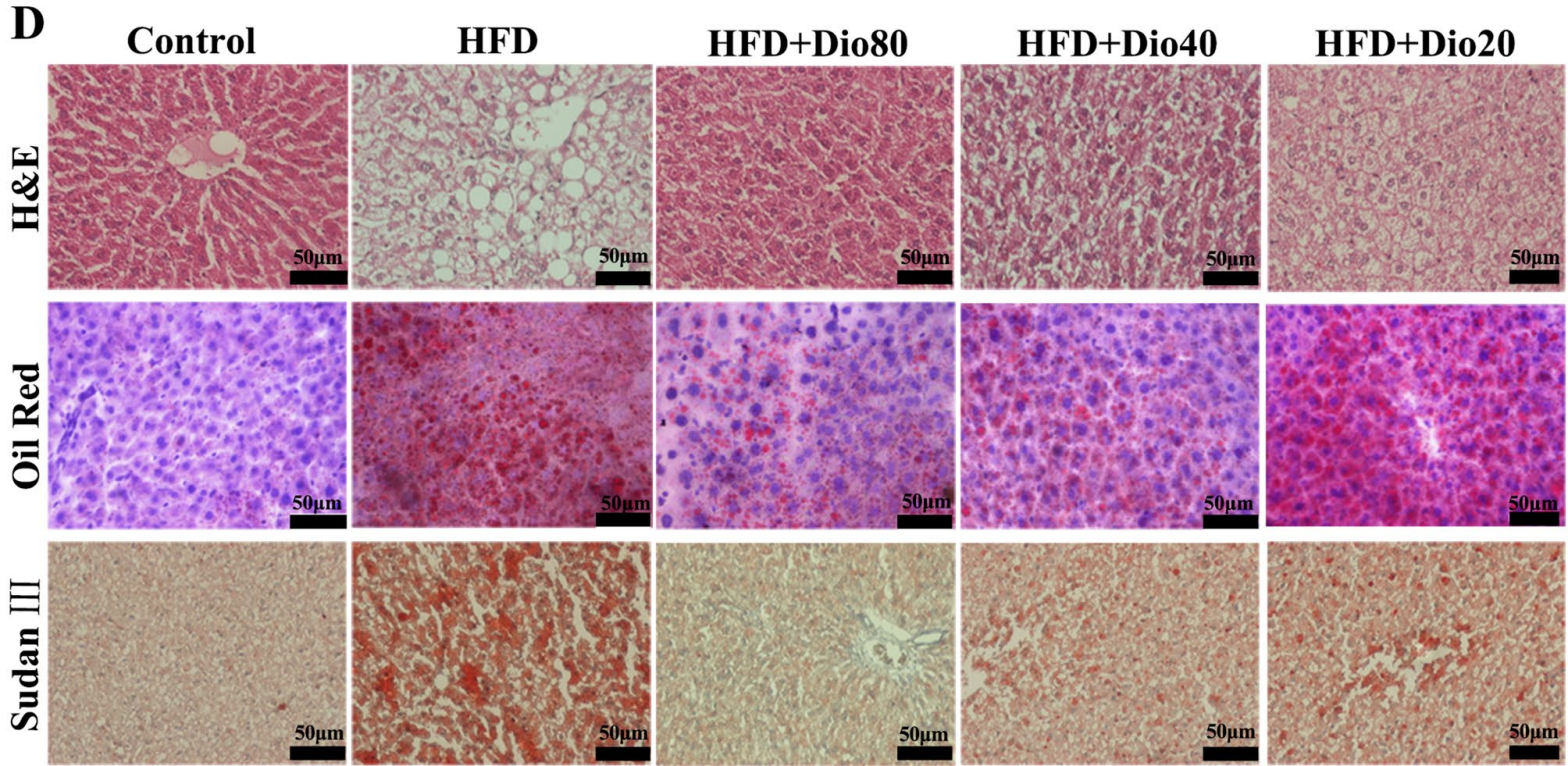
A corrected version of Figure 1D in the Article.

